# The life sulfuric: microbial ecology of sulfur cycling in marine sediments

**DOI:** 10.1111/1758-2229.12538

**Published:** 2017-05-05

**Authors:** Kenneth Wasmund, Marc Mußmann, Alexander Loy

**Affiliations:** ^1^ Department of Microbiology and Ecosystem Science, Division of Microbial Ecology, Research Network “Chemistry meets Microbiology” University of Vienna Althanstrasse 14 Vienna A‐1090 Austria; ^2^ Austrian Polar Research Institute Vienna Austria

## Abstract

Almost the entire seafloor is covered with sediments that can be more than 10 000 m thick and represent a vast microbial ecosystem that is a major component of Earth's element and energy cycles. Notably, a significant proportion of microbial life in marine sediments can exploit energy conserved during transformations of sulfur compounds among different redox states. Sulfur cycling, which is primarily driven by sulfate reduction, is tightly interwoven with other important element cycles (carbon, nitrogen, iron, manganese) and therefore has profound implications for both cellular‐ and ecosystem‐level processes. Sulfur‐transforming microorganisms have evolved diverse genetic, metabolic, and in some cases, peculiar phenotypic features to fill an array of ecological niches in marine sediments. Here, we review recent and selected findings on the microbial guilds that are involved in the transformation of different sulfur compounds in marine sediments and emphasise how these are interlinked and have a major influence on ecology and biogeochemistry in the seafloor. Extraordinary discoveries have increased our knowledge on microbial sulfur cycling, mainly in sulfate‐rich surface sediments, yet many questions remain regarding how sulfur redox processes may sustain the deep‐subsurface biosphere and the impact of organic sulfur compounds on the marine sulfur cycle.

## Introduction

Marine sediments are dynamic environments that are shaped by interactions among biotic and abiotic processes including the redox reactions by which microorganisms harness energy (Schrenk *et al*., 2010). While macrofauna may exist and cause some bioturbation in the most upper sediment layers (Bertics and Ziebis, 2010), the vast diversity and biomass of life at and below the seafloor is predominantly microscopic. Sedimentary microorganisms utilise various combinations of available electron donors and acceptors for energy conservation, the combinations of which are largely under thermodynamic controls (Jørgensen, 2000) and are also highly dependent on the amounts, types and rates of their respective inputs. Together, these factors manifest in the depth stratification of marine sediments i.e. a continuum of more or less overlapping biogeochemical zones, whereby each zone is characterised by the prevailing electron acceptors (Canfield and Thamdrup, 2009). In particular, sulfate is an ubiquitous electron acceptor in marine sediments due to its high concentration in seawater (∼28 mM). Seawater diffuses into sediments and sulfate respiration is thus one of the most important microbial redox process in marine sediments once more energetically favourable electron acceptors such as oxygen, nitrate/nitrite, and iron and manganese oxides are depleted.

**Figure 1:**
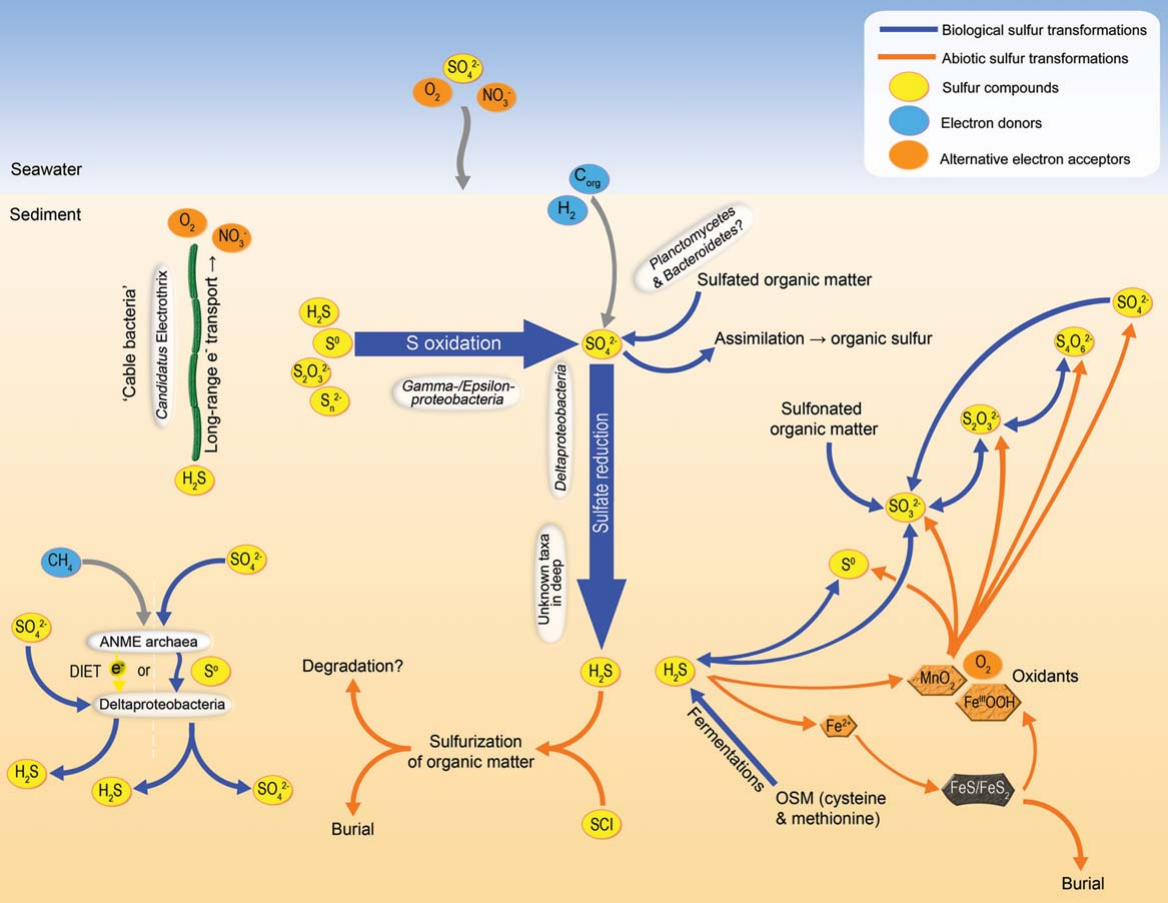
Conceptual depiction of the sulfur cycle in marine sediments, including main reactions of inorganic and organic sulfur compounds, selected taxa, sulfur oxidation via long‐range electron transport by cable bacteria, sulfate‐dependent anaerobic methane oxidation, and transformations of sulfur compounds of intermediate oxidation states (sulfur cycle intermediates, SCI). Blue lines depict biologically‐mediated sulfur transformations that can also be components of disproportionation reactions. Orange lines depict abiotic reactions. Inorganic sulfur compounds are depicted within yellow eclipses. Other electron acceptors are depicted within orange ellipses, and electron donors are depicted within blue ellipses. OSM = organo‐sulfur molecules, C_org_ = organic matter. DIET = direct‐interspecies electron transport. ANME = anaerobic methane‐oxidising.

On a global scale, recent estimates suggest the remineralisation of up to 29% of the organic matter that is deposited to the seafloor is facilitated by sulfate‐reducing microorganisms (SRM) (Bowles *et al*., 2014), which are conventionally regarded as terminal components of anaerobic microbial food webs that cooperatively degrade organic matter. The activity of SRM is particularly important in organic‐rich sediments underlying the highly productive waters of continental shelves and slopes (Jørgensen, 1982; Jørgensen and Kasten, 2006). Global estimates indicate that 11.3 teramoles of sulfate are reduced to hydrogen sulfide in marine sediments every year (Bowles *et al*., 2014). In turn, hydrogen sulfide and other reduced sulfur compounds serve as electron donors for sulfur‐oxidising microorganisms (SOM) or are abiotically oxidised. Sulfate reduction is thereby the primary driver of the biogeochemical cycling of sulfur in marine sediments (Fig. 1). Sulfur cycling is a major determinant of the biogeochemistry and microbial ecology in sulfate‐rich surface sediments and the underlying sulfate‐methane transition zone (SMTZ). Interestingly, the discovery of cryptic sulfur cycling i.e. the rapid recycling of sulfur species at low sulfate concentrations (Holmkvist *et al*., 2011; Brunner *et al*., 2016) and continuous detection of functional genes of SRM (Leloup *et al*., 2007; Leloup *et al*., 2009; Blazejak and Schippers, 2011; Aoki *et al*., 2015) in sulfate‐poor sediment zones deep below the SMTZ revealed that the impact of microbial sulfur metabolism extends to biogeochemical zones that were traditionally viewed as largely fermentative and methanogenic (Bowles *et al*., 2014; Glombitza *et al*., 2016).

Our understanding of sulfur cycling processes and the biology of microorganisms that catalyse them has improved considerably during recent years. Yet, there still remain significant questions regarding the biology of microorganisms and factors that control the turnover of sulfur compounds in marine sediments. For example, literally only the (sediment) surface of the species richness of sulfur‐transforming microorganisms in the subseafloor realm, which is one of the largest microbial biomes on Earth, has been explored. Cycling between the most oxidised (+6, sulfate) and most reduced (–2, sulfide) states of sulfur involves several inorganic sulfur compounds of intermediate oxidation states (i.e. sulfur cycle intermediates, SCI) that allow for (i) sulfur metabolite handoff to other microorganisms and (ii) shortcuts in the sulfur cycle (Fig. 1) (Jørgensen, 1990). The extent by which sulfur cycling in the different biogeochemical sediment horizons is mediated by ‘sulfur compound syntrophy’, i.e. the interspecies transfer of SCI, and the interplay between generalists that utilise diverse sulfur compounds of various oxidation states and specialists that utilise only selected sulfur compounds, is unknown. Additionally, previous research has concentrated on cycling of inorganic sulfur compounds, leaving the impact of organic sulfur compounds on life in the seafloor largely unexplored. Furthermore, individual microorganisms have evolved various, sometimes seemingly redundant enzyme systems to catalyse the many possible conversions of sulfur compounds between different oxidation states. While progress in the biochemical characterisation of these enzymes is continuously made (Denger *et al*., 2014; Felux *et al*., 2015; Santos *et al*., 2015; Johnston *et al*., 2016), some sulfur pathways and sulfur‐converting enzymes are yet to be discovered and/or functionally characterised (Milucka *et al*., 2012).

In this review, we highlight recent and selected findings in marine sediment sulfur cycle microbiology and address knowledge gaps that might become emphases for future research. The review especially aims to tie‐together a perspective on various microbial ecological aspects and on biogeochemical impacts of several key aspects of the sulfur cycle in marine sediments. In some points where little is known about the associated microbial ecology, we point‐out the biogeochemical aspects that suggest their probable key influences on associated microbial ecology. The review is primarily restricted to dissimilatory metabolisms used for energy conservation.

## Dissimilatory sulfate reduction

Despite being of polyphyletic origin and having diverse physiological adaptations, described SRM are unified by having the same central pathway for sulfate respiration with the core enzymes ATP sulfurylase (Sat), adenylyl‐sulfate reductase (Apr), and dissimilatory (bi)sulfite reductase (Dsr) (Table 1) [for a comprehensive overview of SRM physiology, see review by (Rabus *et al*., 2015)]. Estimates of quantities in marine sediments suggest that SRM account for a large proportion (approx. 5–25%) of the microbial biomass in sulfate‐rich zones near the surface, and may have even higher relative abundances in the SMTZ (up to approx. 30–35%) (Leloup *et al*., 2007; Leloup *et al*., 2009). Available substrates and *in situ* temperatures are the key determinants of SRM community structure in sediment zones with sufficient sulfate (Robador *et al*., 2016). Various SRM that are ecologically relevant in these marine sediment layers belong to the class *Deltaproteobacteria*, whereby members of the family *Desulfobacteraceae* are typically one of the most important taxonomic groups in terms of abundance and activity (Fig. 1) (Robador *et al*., 2016).

**Table 1:** Selected sulfur compound‐transforming enzymes and proteins used by microorganisms for dissimilation of sulfur compounds.

General description	Gene/s	Enzyme/protein/complex [cofactor/'type'] ^a^	Sulfur transformation ^b^	Physiological function/s	Reference	UniProt ID ^c^
**Sulfate reduction; sulfur oxidation; other pathways in which sulfite is made available**	*dsrAB*	Dissimilatory (bi)sulfite reductase, subunits AB [siroheme]	sulfite + [DsrC protein]‐dithiol → a [DsrC]‐trisulfide	Key enzyme in canonical sulfate/sulfite reduction; reverse function in sulfur oxidation; present in some non‐sulfate/sulfite‐reducing syntrophs	Santos *et al*. ( 2015)	P45574
	*dsrC*	DsrC	Co‐substrate for DsrAB in sulfite reduction	Acts as a co‐substrate for sulfite reduction by DsrAB	Santos *et al*. ( 2015), Oliveira *et al*. ( 2008), and Venceslau *et al*. ( 2014)	P45573
	*dsrMKJOP*	DsrMKJOP	[DsrC]‐trisulfide → hydrogen sulfide + [DsrC protein]‐dithiol + 2 electron‐transfer quinone	Reduction of DsrC trisulfide, thereby linking cytoplasmic reduction of sulfite to energy conservation at membrane; reverse function in sulfur oxidation	Grein *et al*. ( 2010)	Q72CJ4
	*dsrN*	DsrN	sirohydrochlorin + L‐glutamine → L‐glutamate + siroamide	Amidation of the siroheme cofactor of DsrAB	Lübbe *et al*. ( 2006)	Q9F2A0
	*dsrD*	DsrD	Probable DNA‐binding	Probable transcriptional regulatory element	Hittel and Voordouw ( 2000)	Q46582
	*sat*	Sulfate adenylyltransferase	sulfate + ATP + H^+^ ⇄ adenosine 5′‐phosphosulfate (APS) + diphosphate	Activation of sulfate with ATP to produce APS with higher redox potential than sulfate itself; also used for assimilation of sulfur from sulfate; reverse function in sulfur oxidation	Gavel *et al*. ( 1998)	Q72CI8
	*aprBA*	Adenylylsulfate (APS) reductase	APS + a reduced electron acceptor ⇄ sulfite + AMP + an oxidised electron acceptor + 2 H^+^	Conversion of APS to sulfite, which then acts as substrate for DsrAB/DsrC; reverse function in sulfur oxidation	Lampreia *et al*. ( 1994)	Q72DT2
	*qmoABC*	Quinone‐interacting membrane‐bound oxidoreductase complex	electron transfer	Probable electron donor/transfer to Apr, linked to menaquinone pool. Appears specific for sulfate reduction pathway, but not sulfite reduction; reverse function in sulfur oxidation	Ramos *et al*. ( 2012)	Q7X167
**Sulfite → Sulfide**	*dsr*	Dissimilatory (bi)sulfite reductase [siroheme]	as described above	The Dsr complex and associated enzymes can also be used directly for sulfite reduction	Santos *et al*. ( 2015)	As above.
	*mccA/sirA*	Sulfite reductase [cytochrome]	sulfite → sulfide	Direct respiratory reduction of sulfite to sulfide	Kern *et al*. ( 2011)	Q7MSJ8
	*asrABC*	Anaerobic sulfite reductase [iron‐sulfur]	sulfite → sulfide	Direct respiratory reduction of sulfite to sulfide	Huang and Barrett ( 1990)	P26474
	*fsr*	F_420_‐dependent sulfite reductase [siroheme]	sulfite → sulfide	Sulfite detoxification/sulfur assimilation	Johnson and Mukhopadhyay ( 2005)	Q58280
**Thiosulfate → Sulfite**	*phsABC* ^d^	Thiosulfate reductase [molybdenum]	thiosulfate ⇄ sulfite	Respiratory reduction of thiosulfate yielding sulfite, which is further reduced (as above) to yield energy; thiosulfate disproportionation; thiosulfate oxidation	Burns and DiChristina ( 2009)	P37600
**Thiosulfate ⇄ Tetrathionate**	*tsdA*	Thiosulfate dehydrogenase [diheme cytochrome]	thiosulfate ⇄ tetrathionate	Thiosulfate oxidation; tetrathionate reduction	Denkmann *et al*. ( 2012)	D3RVD4
	*otr*	Octoheme tetrathionate reductase [octoheme cytochrome]	tetrathionate ⇄ thiosulfate	Respiratory reduction of tetrathionate; more efficient as tetrathionate reductase than thiosulfate oxidase; possible role as nitrite reductase	Mowat *et al*. ( 2004)	Q8E9W8
	*ttrABC*	Tetrathionate reductase [molybdenum]	tetrathionate ⇄ thiosulfate	Respiratory reduction of tetrathionate to thiosulfate; some trithionate reductase activity	Hensel *et al*. ( 1999)	Q9Z4S6
	*tetH*	Tetrathionate hydrolase	tetrathionate ⇄ thiosulfate + sulfate	Tetrathionate oxidation	Kanao *et al*. ( 2007)	F9ZNI0
**Thiosulfate → Thiocyanate**	*rdlA*	Rhodanese‐like protein [rhodanase]	thiosulfate + cyanide ‐> thiocyanate + sulfite	Possible role in respiratory thiosulfate reduction; cyanide detoxification	Ravot *et al*. ( 2005)	Q6Q1E2
**Elemental sulfur/polysulfide transformations**	*psrABC*	Polysulfide reductase [molybdenum]	polysulfide → sulfide	Respiratory reduction of polysulfides to sulfide	Krafft *et al*. ( 1992)	P31075
	*hydDACB*	Sulfhydrogenase/hydrogenase I [flavoprotein]	S° or polysulfide → sulfide	Reduction of polysulfides and/or elemental sulfur to sulfide; NADPH oxidation forming H_2_ when sulfur is absent; H_2_ oxidation	Ma *et al*. ( 1993)	Q8U2E5
	*shyCBDA*/*suDH*	Sulfhydrogenase/hydrogenase II (a.k.a. Sulfide dehydrogenase) [flavoprotein]	S° or polysulfide → sulfide	Reduction of polysulfides and/or elemental sulfur to sulfide; NAD(P)H oxidation forming H_2_; H_2_ oxidation; ferredoxin:NADP oxidoreductase	Ma *et al*. ( 2000)	E7FHN9
	*npsr*	NADH‐dependent persulfide reductase [flavoprotein/rhodanase]	S° or polysulfide → sulfide	Not clear; Reduction of range of disulfide, persulfide, and polysulfide compounds and/or elemental sulfur to sulfide	Warner *et al*. ( 2011)	A3QAV3
**Sulfite oxidation**	*soeABC*	Sulfite oxidising enzyme [molybdenum]	sulfite → sulfate	Sulfite oxidation to sulfate; sulfite detoxification after liberation from organo‐sulfur molecules	Lenk *et al*. ( 2011)	D3RNN8
	*sorAB*	Sulfite:acceptor oxidoreductase/Sulfite oxidising enzyme [molybdenum]	sulfite → sulfate	Sulfite oxidation to sulfate, sulfite may be formed during thiosulfate or sulfide oxidation; sulfite detoxificaton after liberation from organo‐sulfur molecules	Kappler *et al*. ( 2000)	Q9LA16
	*sorT*	Sulfite:acceptor oxidoreductase/Sulfite oxidising enzyme [molybdenum]	sulfite → sulfate	Sulfite oxidation to sulfate, sulfite may be formed during thiosulfate or sulfide oxidation; sulfite detoxification after liberation from organo‐sulfur molecules	Wilson and Kappler ( 2009)	M4MVJ1
**Sulfur oxidations**	*sqr*	Sulfide:quinone‐oxidoreductase [flavoprotein]	sulfide → S^0^, ${\text{HS}}_{n}^{–}$ + reduced quinol	Sulfide oxidation; resulting S° or ${\text{HS}}_{n}^{–}$, which are oxidised further by reverse‐Dsr or Sox pathways	Schütz *et al*. ( 1997)	Q4W5U9
	*fccAB*	Flavocytochrome c/sulfide dehydrogenases [flavoprotein/cytochrome]	sulfide → S^0^, ${\text{HS}}_{n}^{–}$	Sulfide oxidation; resulting S° or ${\text{HS}}_{n}^{–}$, which are oxidised further by reverse‐Dsr or Sox pathways	Dolata *et al*. ( 1993)	Q06529
	*soxXA*	Sulfur‐oxidising multi‐enzyme complex, subunits XA [cytochrome]	thiosulfate or sulfite + SoxY → [SoxY protein]‐thiocysteine‐S‐sulfate	Mediates binding of thiosulfate or sulfite to a cysteine residue of SoxY	Bamford *et al*. ( 2002)	Q9LCV0, O33434
	*soxYZ*	Sulfur‐oxidising multi‐enzyme complex, subunits YZ	thiosulfate or sulfite + SoxY → [SoxY protein]‐thiocysteine‐S‐sulfate	Sulfur binding/carrier to form sulfur anion adducts resulting in SoxY‐thiocysteine‐S‐sulfur	Quentmeier *et al*. ( 2003)	Q9LCU9, Q9LCU8
	*soxB*	Sulfur‐oxidising multi‐enzyme complex, subunit B; Sulfate thioesterase/sulfate thiohydrolase [di‐manganese]	[SoxY protein]‐thiocysteine‐S‐sulfate → [SoxY protein]‐S‐thiocysteine + sulfate	Hydrolyses sulfonate moiety of SoxY‐thiocysteine‐S‐sulfate or SoxY‐cysteine‐S‐sulfate, releasing sulfate	Quentmeier and Friedrich ( 2001)	P72177
	*soxCD*	Sulfur‐oxidising multi‐enzyme complex, subunits CD; Sulfane‐sulfur dehydrogenase [molybdenum/cytochrome]	[SoxY protein]‐*S*‐thiocysteine → a [SoxY‐protein]‐L‐cysteine‐*S*‐sulfate + 6 reduced *c*‐type cytochrome	Successive oxidation of outer sulfur of SoxY‐S‐thiocysteine, releasing 6 electrons to cytochromes	Bardischewsky *et al*. ( 2005)	A1B9M5, O07819
	*soxL*	Sulfur‐oxidising multi‐enzyme complex, rhodanese‐like protein	sulfur transfer and trafficking	Possible role in transfer of SoxY‐bound sulfane sulfur to zero‐valent sulfur	Welte *et al*. ( 2009)	D3RVS9
	*rhd‐tusA‐dsrE2*	Rhodanase‐like protein‐Sulfurtransferase	sulfur transfer and trafficking	Elemental sulfur trafficking network from periplasm to cytoplasm for reverse Dsr oxidation; globules of zero‐valent sulfur may also form as intermediates during oxidation of sulfide, polysulfides, elemental sulfur, and thiosulfate to sulfate.	Stockdreher *et al*. ( 2012)	D3RPB9‐D3RPC0‐D3RPC1
	*dsrEFH*	Sulfurtransferase	sulfur transfer and trafficking	Possible sulfur donor to DsrC during sulfur oxidation	Stockdreher *et al*. ( 2014)	O87896‐O87897‐O87898
	*sgpABCD*	sulfur globule proteins	sulfur storage	Envelope formation and expansion of zero‐valent sulfur globules	Weissgerber *et al*. ( 2014), Prange *et al*. ( 2004)	
**Organo‐sulfur transformations**		Sulfatase, a.k.a. sulfuric ester hydrolase ^e^	R‐ ${\text{OSO}}_{3}^{–}$ → ROH + ${\text{SO}}_{4}^{2–}$; RN(H) ${\text{SO}}_{3}^{–}$ → RNH_2_ + ${\text{SO}}_{4}^{2–}$	Removal of sulfate moiety from organic molecules in order to: access organic molecule; obtain sulfur for assimilation; obtain sulfur for respiration	Barbeyron *et al*. ( 2016)	n.a.
	*FGly‐sulfatase*	formylglycine‐dependent sulfhydrolase, sulfatase [formylglycine]	R‐ ${\text{OSO}}_{3}^{–}$ → ROH + ${\text{SO}}_{4}^{2–}$; RN(H) ${\text{SO}}_{3}^{–}$ → RNH_2_ + ${\text{SO}}_{4}^{2–}$	Removal of sulfate moiety from organic molecules in order to: access organic molecule; obtain sulfur for assimilation; obtain sulfur for respiration	Hanson *et al*. ( 2004)	O69787
	*atsK*	alkylsulfodioxygenase [dioxygenase]	R‐ ${\text{OSO}}_{3}^{–}$ → ROH + ${\text{SO}}_{4}^{2–}$; RN(H) ${\text{SO}}_{3}^{–}$ → RNH_2_ + ${\text{SO}}_{4}^{2–}$	Removal of sulfate moiety from organic molecules in order to: access organic molecule; obtain sulfur for assimilation; obtain sulfur for respiration	Kahnert and Kertesz ( 2000)	P9WKZ1
	*sdsA1*	alkylsulfohydrolase	R‐ ${\text{OSO}}_{3}^{–}$ → ROH + ${\text{SO}}_{4}^{2–}$; RN(H) ${\text{SO}}_{3}^{–}$ → RNH_2_ + ${\text{SO}}_{4}^{2–}$	Removal of sulfate moiety from organic molecules in order to: access organic molecule; obtain sulfur for assimilation; obtain sulfur for respiration	Davison *et al*. ( 1992)	Q9I5I9
	*atsA*	arylsulfohydrolase	R‐ ${\text{OSO}}_{3}^{–}$ → ROH + ${\text{SO}}_{4}^{2–}$; RN(H) ${\text{SO}}_{3}^{–}$ → RNH_2_ + ${\text{SO}}_{4}^{2–}$	Removal of sulfate moiety from organic molecules in order to: access organic molecule; obtain sulfur for assimilation; obtain sulfur for respiration	Barbeyron *et al*. ( 1995)	P25549
	*xsc*	sulfoacetaldehyde acetyltransferase	sulfoacetaldehyde → sulfite	Desulfonation of sulfoacetaldehyde in anaerobic sulfolactate degradation; part of taurine degradation pathway; sulfolactate is product of anaerobic sulfoquinovose degradation	Denger *et al*. ( 2009), Felux *et al*. ( 2015)	A3SR25
	*suyAB*	sulfolactate sulfo‐lyase	3‐sulfolactate → sulfite + pyruvate	Desulfonation of 3‐sulfolactate, which can be an intermediate L‐cysteate degradation.	Rein *et al*. ( 2005)	Q58Y43‐Q58Y44
	*cuyA*	cysteate sulfo‐lyase	L‐cysteate → sulfite	Desulfonation of cysteate in sulfolactate degradation pathway	Denger *et al*. ( 2009)	A3SQG3
	*tauD*	taurine dioxygenase	taurine → sulfite + 2‐aminoacetaldehyde + succinate + CO_2_	Expressed only under conditions of sulfate starvation, aerobic	van der Ploeg *et al*. ( 1996)	P37610

This table is intended to provide an overview of enzymes catalysing key reactions in dissimilatory sulfur metabolisms described in the text and may aid in developing new marker genes for microbial ecological studies and interpreting (meta)genome/transcriptome data.

^a^We provide a very brief keyword regarding cofactors in order to help distinguish different enzyme types.

^b^In some cases ions and oxidizing/reducing agents are omitted for simplicity.

^c^UniProt IDs are mainly listed for substrate‐specific catalytic subunits.

^d^Molybdenum‐type oxidoreductases: Alpha subunit is catalytic, Beta subunit is electron transfer from Gamma subunit, Gamma subunit is membrane anchor.

^e^Four sub‐classes are listed directly below.

a.k.a = also known as.

n.a. = not available.

Prototypical of SRM in general, marine *Desulfobacteraceae* may use major fermentation products such as hydrogen, formate, acetate, and propionate as electron donors for sulfate respiration, but may also forage on a variety of other substrates including longer fatty acids, alkanes, and aromatic compounds, some of which allow these organisms to thrive at natural hydrocarbon seeps (Kleindienst *et al*., 2014; Na *et al*., 2015; Dorries *et al*., 2016b). Detailed proteomic‐genomic analyses of cultured representatives of the marine *Desulfobacteraceae* indicated their flexibility and ecological success is afforded by a combination of physiological mechanisms (Dorries *et al*., 2016a, 2016b). These include multiple oxygen defence systems, various signal transduction pathways for sensing different environmental cues such a substrate availability, as well as abundant and constitutively expressed membrane‐bound redox complexes that are important for linking electron flows from the catabolism of different substrates to respiration. Recent studies suggest that members of the uncultured Sva0081 clade of the *Desulfobacteraceae* are particularly abundant in coastal surface sediments around the world. They appear especially adapted to deal with oxidative stress from oxygen penetration in shallow sediments and may be exceptionally metabolically flexible, as inferred from their very large genomes (e.g. up to 9 Mb) (M. Mussmann, unpubl. data). However, *Desulfobacteraceae* represent just the iceberg tip of the SRM taxon diversity in marine sediments.

**Figure 2:**
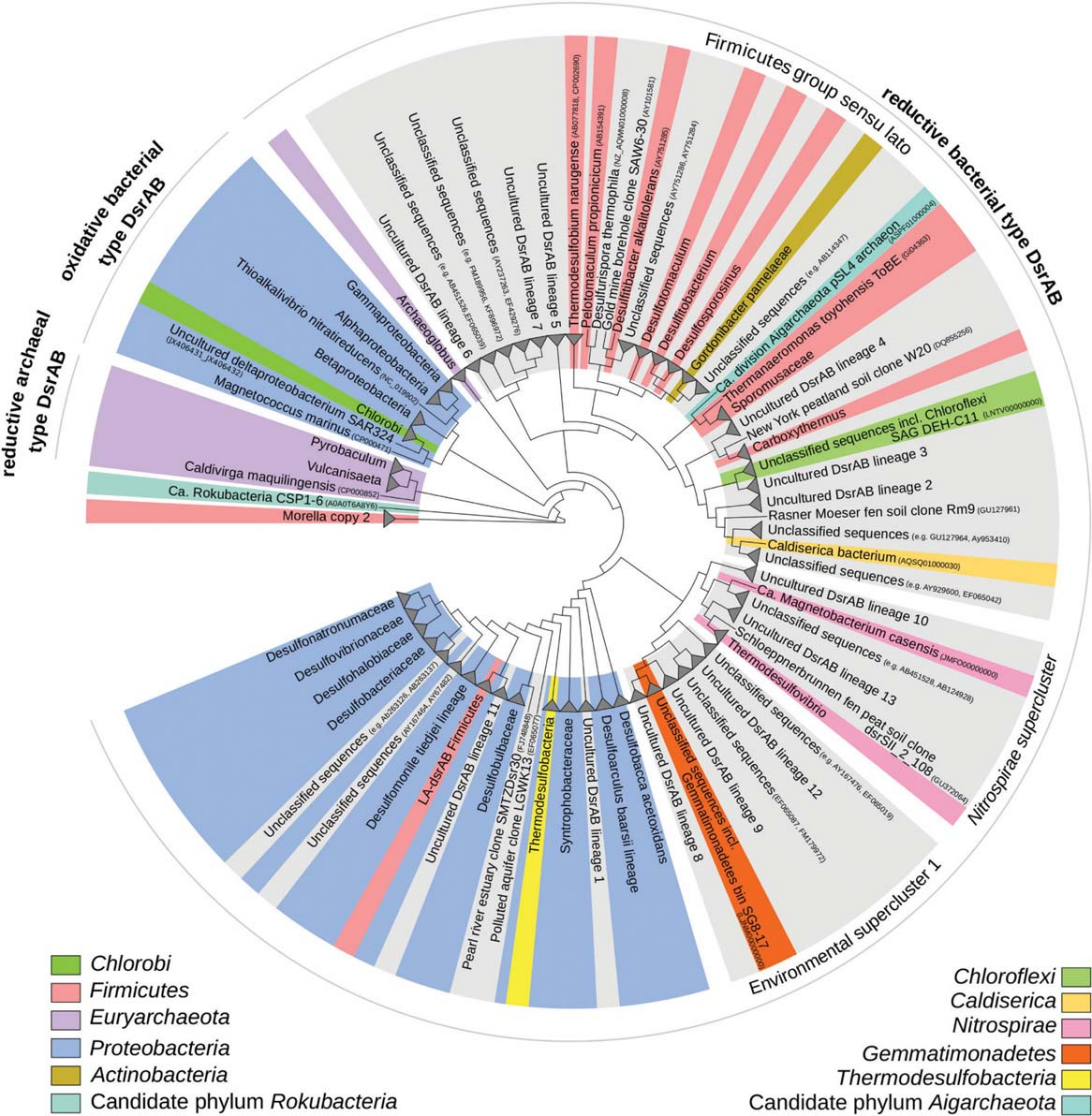
Phylogenetic tree showing the diversity of major DsrAB lineages, including sequences from the environment and isolated strains. The tree was based on a previously described DsrAB sequence set (Müller *et al*., 2015), including new sequences from the phyla *Chloroflexi* (Wasmund *et al*., 2016) and *Gemmatimonadetes* (Baker *et al*., 2015), and the candidate phylum *Rokubacteria* (Hug *et al*., 2016), and constructed with Fasttree (LG model of amino‐acid evolution) and an indel filter covering 530 alignment positions. Branches are unscaled. Clades with representatives from known phyla are labelled in different background colours. *Desulfatiglans anilini* lineage (not shown) is collapsed into the LA‐dsrAB Firmicutes group. Clades without cultured representatives are shown in grey. The three major DsrAB protein families, namely the reductive bacterial type, the oxidative bacterial type and the reductive archaeal type, are shown. The designation ‘uncultured DsrAB lineage’ depicts a stable, monophyletic lineage that consists only of environmental *dsrAB* sequences and has family‐level or higher taxon diversity. LA‐*dsrAB*, laterally acquired *dsrAB*. *Moorella dsrAB* copy 1 clustered with the LA‐*dsrAB Firmicutes* group. Selected accession numbers (for screen‐view only due to small font size) are given for some branches to aid in identification. See publication by Müller *et al*. (2015) for further information.

Other known key taxa include the families *Desulfobulbaceae* and *Syntrophobacteraceae*, and a wide diversity of bacteria related to the genus *Desulfatiglans*, which likely represents a previously undescribed deltaproteobacterial family. More astonishing is the extraordinary, yet undescribed diversity of SRM that is only recognised to exist from molecular ecology surveys of functional marker genes for SRM, i.e. *aprBA* and *dsrAB* (Müller *et al*., 2015). Marine *dsrAB*‐containing microorganisms are affiliated with several major uncultivated DsrAB lineages of family‐level or even higher taxon diversity, of which some (e.g. DsrAB lineages 2, 3, and 4) are almost exclusively found in marine ecosystems (Fig. 2) (Müller *et al*., 2015). Recent applications of single‐cell genomics and metagenomics to marine sediments have only just begun to uncover the phylogenetic identities of such *dsrAB*‐containing microorganisms. For instance, *dsrAB* sequences related to the marine‐specific DsrAB lineages 2, 3 and 4, have been uncovered in members of the *Chloroflexi*, which are notable for being relatively abundant in deep sediments and could therefore hint to their roles in the deep subsurface sulfur cycle (Wasmund *et al*., 2016). Additionally, a complete core gene set for sulfate reduction, including *dsrAB* from an uncultivated lineage, was recently discovered in a genome bin (SG8‐17) of a member of the phylum *Gemmatimonadetes* that was recovered from a sulfate‐rich estuarine sediment layer (Fig. 2) (Baker *et al*., 2015). Although promising, there is a long way ahead in charting the taxon diversity of marine SRM and sulfur‐transforming microorganisms by environmental genomics approaches due to the expansive microbial diversity found in marine sediments.

Our understanding of the ecology and lifestyles of SRM beyond the SMTZ and in the deep biosphere is even more limited. Sulfate depletion, decreasing nutrient availability and increasing pressure and temperature are general environmental factors that constrain the assembly of SRM communities in deeper sediment layers (Glombitza *et al*., 2015; Roussel *et al*., 2015). Biogeochemical data suggests the existence of high‐affinity SRM that are especially adapted to low sulfate concentrations in marine sediments (Tarpgaard *et al*., 2011). ‘Hot zones’ of cryptic sulfur cycling might provide opportunities for sulfate reduction, but SRM may also have to increasingly rely on their alternative metabolic capabilities such as fermentation due to decreased substrate availability in the deep (Glombitza *et al*., 2016). Metabolic versatility (Plugge *et al*., 2011) and longevity, enabled by cellular resistance mechanisms such as spore formation (Aüllo *et al*., 2013), will enable some SRM to thrive or at least survive during sediment burial (Hubert *et al*., 2009). Nevertheless, SRM communities present in sulfate‐rich and sulfate‐poor zones differ considerably (Leloup *et al*., 2009), but which SRM are active (Orsi *et al*., 2016), and how they make living in the deep remain key research questions.

Besides SRM ecology, recent research has also lead to unexpected discoveries regarding the biochemistry of sulfate reduction. One of the last conundrums of this pathway, namely how energy is conserved during reduction of (bi)sulfite to sulfide, was solved by showing that the DsrC protein is a co‐substrate for DsrAB (Santos *et al*., 2015). Furthermore, it has been an accepted fact that all SRM use the same sulfate reduction pathway. Hence, the possible existence of another pathway for sulfate reduction, which was identified by studying an anaerobic microbial consortium that oxidised methane in a sulfate‐dependent manner, came as a surprise (Milucka *et al*., 2012). It was shown that the methanotrophic archaea, known to lack the canonical sulfate reduction pathway, themselves reduced sulfate, albeit not to hydrogen sulfide but to elemental sulfur. The produced zero‐valent sulfur was then disproportionated to disulfide and sulfate by the associated deltaproteobacterium, which intriguingly harbour the canonical sulfate reduction pathway. It will be exciting to see how widespread and environmentally relevant this alternative pathway for dissimilatory sulfate reduction is, once its genetic and biochemical basis is unraveled and provides the necessary biomarkers for environmental surveys.

## Anaerobic oxidation of methane coupled to sulfate reduction

Natural gases, mostly methane but also other alkanes such as ethane, propane, and butane (Laso‐Perez *et al*., 2016), originate from deep marine sediments or reservoirs in the subseafloor and diffuse upwards. Yet, teragrams of methane per year do not reach the water column because of its oxidation in anoxic sediment zones with sulfate as the dominant electron acceptor. So far, it was assumed that sulfate‐coupled methane oxidation is catalysed by an anaerobic consortium of methanotrophic archaea (ANME archaea) and sulfate‐reducing *Deltaproteobacteria* and that the basis of this syntrophy is the interspecies exchange of a hypothetical diffusible metabolite such as hydrogen, formate or methanethiol (Meulepas *et al*., 2010). A series of recent publications has now provided radically new insights into the biology of these anaerobic consortia, suggesting that they evolved diverse mechanisms for sulfate‐dependent methane oxidation (Fig. 1). As aforementioned, one such mechanism is that the methanotrophic archaea themselves perform sulfate reduction to zero‐valent sulfur (Milucka *et al*., 2012). The deltaproteobacterial partner would thus not be essential, rather a commensal that profits by disproportionation of the produced zero‐valent sulfur compounds. Another possible mechanism is that both partners fulfil their originally proposed function, i.e. the archaeon oxidises methane to CO_2_ and the deltaproteobacterium reduces sulfate to sulfide, but reducing equivalents are exchanged by direct cell‐to‐cell contacts between the partners and not via a diffusible metabolite (McGlynn *et al*., 2015; Wegener *et al*., 2015; Krukenberg *et al*., 2016). Electrical conductivity modelling and experimental data, including expression of large multi‐haem cytochromes by both partners, expression of type IV pili by the deltaproteobacterium, redox‐dependent staining of the intercellular matrix, and nanowire‐like structures between cells, provided evidence of direct interspecies electron transfer (DIET) (Wegener *et al*., 2015). Furthermore, for the first time the anaerobic methanotrophic archaea could be experimentally decoupled from syntrophic SRB by using soluble artificial single electron acceptors such as 9,10‐anthraquinone‐2,6‐disulfonate, humic acids, or Fe(III) complexes (Scheller *et al*., 2016). This finding is further proof for DIET and suggested a mechanism for methane oxidation coupled to insoluble iron(III) and manganese(IV) reduction, namely that methanotrophic archaea respire solid abiotic oxidants (such as metals) directly via extracellular electron transfer.

## Dissimilatory reduction of sulfur cycle intermediates

As described above, the dissimilatory reduction of sulfate drives the formation of enormous quantities of reduced sulfide in marine sediments. Interestingly, these sulfides are subject to an array of biological and chemical oxidation reactions that may result not only in the reformation of sulfate, but also in a number of other SCI. These compounds may include sulfite, elemental sulfur, polysulfides, and polythionates such as thiosulfate and tetrathionate (Fig. 1). Indeed, biogeochemical studies suggest most sulfide (80–90%) is eventually reoxidised, while only 10–20% is ultimately buried, e.g. as complexes with iron (i.e. FeS, FeS_2_) or with organic matter after sulfurization reactions (Berner, 1982; Jørgensen, 1982; Brüchert, 1998; Sinninge Damsté *et al*., 1988; Ferdelman *et al*., 1999). Chemical oxidants of biogeochemical significance may include oxygen, nitrate, manganese‐oxides, iron‐oxides and even organics, which are present to varying degrees at different sediment sites and depths, depending on inputs and fluxes of both organics and inorganics (Burdige and Nealson, 1986; Canfield, 1989; Yao and Millero, 1996; Heitmann and Blodau, 2006; Yu *et al*., 2015). Intriguingly, these SCI can also be utilised as electron donors, as terminal electron acceptors for respiration, or as a both in disproportionation metabolisms (the later are described further below).

In most marine sediments examined, SCI are generally only measurable in the low micromolar ranges and do not accumulate to high concentrations because they are rapidly utilised or react upon formation, e.g. turnover times of few hours have been measured for micromolar additions of thiosulfate and tetrathionate (Jørgensen, 1990; Thamdrup *et al*., 1994; Zopfi *et al*., 2004). For other SCI such as sulfite or elemental sulfur, accessibility may hinder their utilisation, for instance, sulfite is particularly reactive with organics or elemental sulfur and it may therefore have short residence times, while elemental sulfur is stable but rather insoluble, which may also hinder its availability to microorganisms (Schauder and Kröger, 1993; Vairavamurthy *et al*., 1994; Zopfi *et al*., 2008). Only sediments with particularly high sulfate reduction rates and therefore high levels of sulfide production, e.g. those associated with particularly high organic loads or salt marsh beds, seem to exhibit accumulations of SCI (Zopfi *et al*., 2004). Additions of thiosulfate to sediments cause drastic decreases in the rates of sulfate reduction (Jørgensen, 1990), suggesting they draw electron‐flows away from specialist SRM to other organisms, or that SRM may switch to the use of SCI when available, or combinations thereof. Interestingly, parallel experiments have shown that tetrathionate additions reduced sulfate reduction rates to a much lesser extent, which suggests organisms fundamentally different to typical SRM may be largely responsible for tetrathionate reduction (Zopfi *et al*., 2004). The rapid turnover of SCI may not be surprising, since compared with sulfate, some SCI have higher redox potentials and/or more energetically favourable pathways. For instance, tetrathionate has a very high redox value of +198 ± 4 mV (Kurth *et al*., 2016); sulfite reduction bypasses the activation step of sulfate, which requires two ATP equivalents, and thus can lead to greater growth yields depending on the electron donor (Badziong and Thauer, 1978; Simon and Kroneck, 2013). Together, these facts mean some SCI are preferentially utilised as respiratory electron acceptors over sulfate. The available biogeochemical data clearly indicates that resident microorganisms in marine sediments are highly adapted and poised for the utilisation of various SCI.

To date, diverse microorganisms are known to be capable of, or have the genetic potential to, respire different and/or multiple SCI (Harel *et al*., 2016). However, this evidence is clearly far from complete, since it is reliant on information from the yet limited number of cultivated organisms or genomic information retrieved from environmental genomics. Nevertheless, in marine sediments, many cultivated *Deltaproteobacteria* are known to respire SCI and harbour SCI‐reductases (Krämer and Cypionka, 1989). In various tested SRM cultures, enzymes for thiosulfate and sulfite transformations were seemingly constitutively expressed (Krämer and Cypionka, 1989). This information may support the notion that SRM switch to SCI when available and why sulfate reduction rates decrease when SCI are added to sediments. Recently, various *Desulfuromonadales* phylotypes were highlighted as probable elemental sulfur respirers (or disproportionators) in diverse benthic habitats (Pjevac *et al*., 2014). Diverse members of the phyla *Proteobacteria* and *Bacteroides*, which are typically found in marine sediments are known to be capable of respiring elemental sulfur (Florentino *et al*., 2016).

Genes for key catalytic enzymes required for SCI transformations, such as tetrathionate‐, thiosulfate‐, polysulfide‐reductases of the complex iron‐sulfur‐molybdenum family of enzymes (Table 1) have been documented in an array of phyla, notably in the *Proteobacteria* and *Firmicutes* (Harel *et al*., 2016). The recent application of genome‐centric metagenomics to marine sediments has revealed potential for sulfur and possibly thiosulfate reduction in taxa completely undescribed and not previously connected with the sulfur cycle, such as the archaeal phylum *Torarchaeota* (Seitz *et al*., 2016; Lazar *et al*., 2017). Future research applying similar culture‐independent genomic approaches will likely illuminate a variety of microbial taxa capable of transforming SCI. Understanding the presence and expression of these genes could be of special interest in marine sediments, since they can act as molecular proxies for transformations of such cryptic biogeochemical processes, and such strategies have proven highly informative for the study of SCI transformations in the water column of oxygen minimum zones, where tracing the actual chemical transformations is difficult (Canfield, 2010). Interestingly, although not studied in marine sediments, enzymes with previously undescribed physiological functions have recently been shown to be capable of reducing SCI, e.g. sulfite, thiosulfate and tetrathionate reductases of the cytochrome family (Table 1) (Kern *et al*., 2011; Simon *et al*., 2011; Liu *et al*., 2013; Kurth *et al*., 2016). It is therefore very probable that the ability to respire SCI is even more widespread than previously conceived.

## Sulfur oxidation

While anaerobic microorganisms respiring sulfur compounds can prevail deep into the marine subsurface, SOM on the other hand are generally restricted to upper sediment layers where electron acceptors with high enough redox potentials for coupling with sulfur oxidation, such as oxygen or nitrate, are available. The biological oxidation of reduced sulfur compounds competes with chemical reactions, in particular with the iron‐driven oxidation of sulfide to FeS or FeS_2_ (Jørgensen and Nelson, 2004) (Fig. 1). However, the half‐life of sulfide in microbial mats is controlled by cell density and is usually lower than in oxic (and iron‐rich) seawater (Nelson *et al*., 1986; Poulton *et al*., 2004; Canfield *et al*., 2005). Generally, thermodynamic and kinetic considerations suggest that biological oxidation probably far exceeds chemical oxidation of sulfide in most environments (Luther *et al*., 2011). While microorganisms are central to sulfide oxidation in marine pelagic oxygen minimum zones (Lavik *et al*., 2009), the general contribution of microorganisms to total sulfur oxidation in marine sediments is still unknown. A major limiting factor for sulfur oxidation in the seafloor are the gradients of oxygen, nitrate and sulfide, which often result in spatially separated maximal concentrations of these potential electron donors and acceptors (Canfield and Thamdrup, 2009). Many SOM therefore cannot directly access their primary energy sources, i.e. sulfides and SCI, and electron acceptors at the same time. This unique evolutionary pressure challenged SOM to develop distinct strategies that are reflected in remarkable morphological adaptations attracting the attention of researchers from different disciplines.

The morphologically conspicuous, large sulfur bacteria (LSB) of the gammaproteobacterial family *Beggiatoaceae* have been model organisms for benthic sulfur oxidation for decades. This family comprises several, morphologically distinct genera with diverse ecological strategies that reflect adaptations to the physicochemical characteristics of a wide spectrum of marine surface sediments (Teske and Salman, 2014). The molecular and morphological characteristics of *Beggiatoaceae* have recently been integrated in a revised taxonomic framework (Salman *et al*., 2011). The LSB are typical ‘gradient organisms’ that are indicative of hypoxic and sulfidic conditions in aquatic sediments. To bridge the spatial gap between oxygen and sulfide they internally store large amounts of the alternative electron acceptor nitrate and of elemental sulfur, a central intermediate of sulfide and thiosulfate oxidation. Filamentous, motile members such as *Candidatus Isobeggiatoa* and *Thioploca* glide between oxic/suboxic and sulfidic sediment layers to replenish their nitrate and sulfur reservoirs, while the non‐filamentous *Thiomargarita* depend on alternating sulfidic and oxygenated resuspensions of sulfidic sediments into oxygenated seawater. Certain *Thiomargarita* and *Thiopilula* attach to rocks or moving shells, where they experience alternating conditions of high sulfide, nitrate and oxygen fluxes (Bailey *et al*., 2011). Genome analysis of single cells or filaments revealed canonical sulfur oxidation pathways and the capacity for denitrification and/or nitrate ammonification (MacGregor *et al*., 2013; Winkel *et al*., 2016). In anoxic sediments, marine *Thioploca* species that respire nitrate to ammonia and leaking nitrite can fuel the anaerobic oxidation of ammonia to N_2_ by associated anammox bacteria and thereby drive a significant nitrogen loss (Prokopenko *et al*., 2013). In contrast, nitrifiers associated with LSB mats can re‐oxidise ammonia to nitrate and may thereby recycle bioavailable nitrogen within LSB mats (Winkel *et al*., 2014). Interestingly, some members of marine LSB such as *Candidatus Thiomargarita nelsonii* may employ both the Calvin‐Bassham‐Benson and the reverse tricarboxylic acid cycle for carbon fixation. Hence, these are currently the only known free‐living bacteria equipped with two carbon fixation pathways (Winkel *et al*., 2016).

Another intriguing evolutionary strategy of SOM to overcome the limited access to oxidants and reductants in marine surface sediments is to partner up with motile, eukaryotic hosts. In such mutualistic, chemosynthetic symbioses, the thioautotrophic bacterium nourishes the host, which in turn provides enhanced access to resources and shelter to the symbiotic SOM. Like the filamentous *Beggiatoaceae*, the motile hosts can position themselves in opposing gradients of oxygen and sulfide or shuttle themselves between oxic/suboxic and sulfidic layers. This lifestyle has developed independently and multiple times between alpha‐ and gammaproteobacterial thioautotrophs and sediment‐dwelling ciliates, oligochaetes, nematodes, flatworms and bivalves [for review see (Dubilier *et al*., 2008)]. Some SOM hosting clams reach high biomasses underneath seagrass meadows and might substantially contribute to sulfide oxidation (van der Heide *et al*., 2012). Intriguingly, these symbiotic SOM are also capable of fixing N_2_ and possibly provide the host not only with carbon, but also with bioavailable nitrogen (Petersen *et al*., 2016).

Recently, an entirely new and rather astonishing mechanism for sulfur oxidation, i.e. electrogenic sulfur oxidation (e‐SOx), was discovered. In this process, multicellular filamentous bacteria bridge the oxidation of sulfide in anoxic sediment layers replete with sulfide to the reduction of oxygen or nitrate in oxic surface sediments, thereby generating and mediating electric currents in marine sediments over centimetre distances (Fig. 1). These so‐called *cable bacteria* are thought to conduct electrons through structures inside a common periplasm of the multicellular filament (Nielsen *et al*., 2010; Pfeffer *et al*., 2012; Marzocchi *et al*., 2014). The spatially separated half‐reactions of e‐SOx result in a characteristic biogeochemical profile, which has strong effects on biogeochemical cycles at aquatic sediment surfaces, for instance, by influencing iron speciation (Seitaj *et al*., 2015; Nielsen, 2016; Sulu‐Gambari *et al*., 2016). The cable bacteria belong to the family *Desulfobulbaceae* of the *Deltaproteobacteria* and are currently represented by two candidate *Genera*, *Candidatus Electronema* and *Candidatus Electrothrix* (Trojan *et al*., 2016). The cable bacteria are widespread in shallow marine sediments (Risgaard‐Petersen *et al*., 2012; Malkin *et al*., 2014; Burdorf *et al*., 2016). *In situ* mass developments of cable bacteria have been documented in a seasonally hypoxic marine basin in the Netherlands (Grevelingen) (Seitaj *et al*., 2015). Similar to the *Beggiatoaceae*, cable bacteria seem to prefer rather diffusive, undisturbed sediments with stable hydrodynamic conditions (Malkin *et al*., 2014; Seitaj *et al*., 2015). The detailed physiology and the genetic background of e‐SOx are still completely unknown and enigmatic, but first experiments with ^13^C‐labelled carbon suggested heterotrophic sulfide oxidation in a marine sediment (Vasquez‐Cardenas *et al*., 2015).

To date, the aforementioned groups of SOM have largely monopolized the attention of scientists studying benthic sulfur oxidation because of their conspicuous morphologies and fascinating lifestyles. However, these occur in high abundances only in certain habitats. In particular, the LSB and cable bacteria seem to be restricted to undisturbed sediment with stable hydrodynamic conditions, while symbiotic SOM and their hosts have been mainly found in permeable coastal sediments. Numerous 16S rRNA gene surveys of marine sediments suggest that virtually all types of marine sediments harbour a myriad of less conspicuous SOM, which probably far exceed the currently known diversity and global abundance of *Beggiatoaceae*, symbiotic SOM and cable bacteria. The unambiguous identification of sulfur oxidation potential in an environmental sample is obscured by the fact that SOM have diverse and complex sulfur oxidation pathways. There is currently no clearly discernible, molecular marker that is universal among all SOM. Sulfide‐quinone oxidoreductase (Sqr) and the thioesterase subunit SoxB of the thiosulfate‐oxidising multienzyme complex (SOX‐pathway) appear to be the most widespread markers among known marine SOM (Table 1), but available primers used for diversity surveys of the respective genes most likely underestimate the actual diversity in marine sediments.

Despite these limitations, the application of functional marker genes in PCR or metagenomic approaches has revealed a yet unseen diversity of SOM. Genes encoding reverse DsrAB, Sqr and SoxB have been amplified from coastal sediments and uncovered a large diversity of alpha‐, gamma‐, and epsilonproteobacterial SOM and also novel, unknown lineages (Pham *et al*., 2008; Lenk *et al*., 2011; Thomas *et al*., 2014). For instance, an uncultured *Rhodobacteraceae* (*Roseobacter*‐clade) member from tidal sediments harboured the reverse DSR‐pathway (Lenk *et al*., 2012). Intriguingly, this SOM possesses both a complete Sox‐ and the reverse DSR‐pathway, and thus a flexibility in sulfur oxidation that is unique among SOM.

Members of the epsilonproteobacterial order *Campylobacterales* occur in virtually all marine systems such as pelagic oxygen minimum zones, hydrothermal systems and marine sediments, typically in habitats with low oxygen/sulfide ratios. They are among the best studied SOM as they often occur in high abundances and are relatively easy to grow in the laboratory. In some highly sulfidic marine sediments, *Arcobacter* species oxidise sulfide to filamentous elemental sulfur forming mat‐like precipitates (Wirsen *et al*., 2002). Members of the *Sulfurovum/Sulfurimonas‐*group seem to be more competitive than other SOM in exploiting solid, elemental sulfur in marine benthic habitats, possibly due to their capability to activate cyclo‐octasulfur (S8) (Pjevac *et al*., 2014). Moreover, *Sulfurimonas* has been repeatedly found in increased frequencies in the rhizosphere of marine plants (Jensen and Kühl, 2007; Thomas *et al*., 2014; Cúcio *et al*., 2016). Interestingly, it has been hypothesised that some *Sulfurimonas* use cable bacteria as an electron sink by DIET when oxygen and nitrate are depleted (Vasquez‐Cardenas *et al*., 2015; Lovley, 2016).

Remarkably, 16S rRNA and functional gene assays have consistently detected relatives of gammaproteobacterial, thiotrophic symbionts of marine protists and invertebrates in high frequencies in marine surface sediments (Ravenschlag *et al*., 1999; Bowman and McCuaig, 2003; Lenk *et al*., 2011; Dyksma *et al*., 2016). Whether these are free‐living or actual symbionts of yet unidentified micro‐ or meiofaunal hosts, is not known, but is certainly an interesting avenue for future research. Moreover, 16S rRNA sequences affiliating with the genera *Sedimenticola*, *Thiohalophilus*, *Thiohalorhabdus*, *Thiomicrospira*, *Thioprofundum* and *Thioalkalivibrio* have been repeatedly detected, but *in situ* abundances of these SOM are still unknown. High relative cell and sequence abundances of members of the recently established family *Woeseiaceae*/JTB255 and of the *Acidiferrobacteraceae* have been detected in sediments worldwide (Bowman *et al*., 2005; Du *et al*., 2016; Dyksma *et al*., 2016). Genomic and isotopic tracer studies confirmed a thioautotrophic potential for members of these families (Dyksma *et al*., 2016; Umezawa *et al*., 2016; Mussmann *et al*., 2017). In particular, the facultative chemolithoautotrophic *Woeseiaceae*/JTB255 are abundant, core members of microbial communities in virtually all studied sediments worldwide, although not all members can oxidise sulfur (Du *et al*., 2016; Mussmann *et al*., 2017). Collectively, the alpha‐, gamma‐ and epsilonproteobacterial SOM may amount to average cell abundances of approximately 10^8^ cells cm^−3^ in organic‐rich marine sediments (Ravenschlag *et al*., 2001; Bowman *et al*., 2005; Lenk *et al*., 2011; Dyksma *et al*., 2016). Given that biotic sulfur oxidation may by far exceed abiotic sulfur oxidation in most habitats and the relatively narrow habitat range of large sulfur bacteria, these inconspicuous SOM possibly account for a major fraction of sulfur oxidation in many marine sediments.

SOM in coastal sediments may also play a central role in marine dark carbon fixation. Recent biogeochemical modelling suggested that sulfur‐dependent carbon fixation in marine sediments could be responsible for almost half of total dark carbon fixation in the oceans (Middelburg, 2011). In line with this, recent isotopic tracer studies found that *Gammaproteobacteria*, including *Acidiferrobacteraceae*, *Woeseiaceae* and others, accounted for a major fraction of CO_2_ fixation in coastal sediments (Boschker *et al*., 2014; Dyksma *et al*., 2016). This raises the possibility that the highly abundant and widespread autotrophic SOM might play a profound, yet unconsidered role in oceanic carbon sequestration (Hawley *et al*., 2014; Dyksma *et al*., 2016).

## Disproportionation of inorganic sulfur compounds

The disproportionation of SCI is performed by various anaerobes that are generally also capable of respiring with sulfur compounds. Disproportionation entails using SCI as both electron donor and acceptor, i.e. an ‘inorganic fermentation’. The biogeochemical importance of these metabolisms was demonstrated by pioneering studies applying radioisotope tracing of different sulfur atoms and demonstrated a significant proportion, i.e. 62–66% and 35–39% of thiosulfate (often the key SCI) is disproportionated in oxidised or reduced sediment layers, respectively (Bak and Cypionka, 1987; Fossing and Jørgensen, 1990; Jørgensen, 1990). While data regarding the biogeochemical impact of the disproportionation of other SCI in marine sediments is still limited, disproportionation of elemental sulfur has been demonstrated (Thamdrup *et al*., 1993). Disproportionation of elemental sulfur must, however, be coupled to scavenging of the resulting sulfide, for example by metal oxides, in order to keep its concentration low and make disproportionation energetically favourable. This may limit its importance to restricted biogeochemical zones containing such scavengers. As described above, disproportionation of zero‐valent sulfur might be the physiology that links the deltaproteobacterial partner to the anaerobic methanotrophic archaea. Although still disputed, recent works have intriguingly proposed that distinct sulfur isotope signatures recorded in ancient marine sediment deposits (3.4 billion‐years‐old) are evidence that sulfur‐disproportionating metabolisms existed and possibly preceded sulfate‐reducing metabolism (Philippot *et al*., 2007; Wacey *et al*., 2011). Numbers of SCI‐disproportionating microorganisms in marine sediments have been estimated by most‐probable number counts, which showed numbers of up 10^7^ cells per cm^3^ sediment capable of disproportionating thiosulfate (Jørgensen and Bak, 1991). Important to note, is that this does not necessarily reflect the *in situ* activity of the populations, because many of these populations are possibly SRM that can switch to disproportionating metabolisms when alternative electron donors/acceptors are lacking.

Many typical sulfur compound‐dissimilating anaerobes have been shown to also disproportionate SCI. These include some members of the deltaproteobacterial family *Desulfobulbaceae* (e.g. *Desulfocapsa*) and the deltaproteobacterial genera *Desulfovibrio* and *Desulfomonile*, the facultative anaerobic gammaproteobacterium *Pantoea agglomerans*, and few members of the phlya *Thermodesulfobacteria* and *Firmicutes* (Mohn and Tiedje, 1990; Finster *et al*., 1998; Jackson and McInerney, 2000; Obraztsova *et al*., 2002; Mardanov *et al*., 2016). Generally, our knowledge of SCI‐disproportionating microorganisms is purely based on cultivated strains, since no functional marker genes/enzymes can be used to unambiguously distinguish these metabolisms from other sulfur dissimilation pathways. Nevertheless, *Desulfocapsa*‐related bacteria can often be detected in marine sediments from various sites and appear to be prevalent in association with seagrasses, possibly reflecting higher amounts of SCI measured in such environments (Sun *et al*., 2015; Cúcio *et al*., 2016). *In situ* incubations combined with molecular surveys of microbial communities colonizing elemental sulfur particles suggested various *Desulfobulbaceae* may disproportionate elemental sulfur (Pjevac *et al*., 2014). Intriguingly, some bacteria (e.g. *Desulfocapsa sulfexigens*, *Thermosulfurimonas dismutans*) appear to be specialised SCI disproportionators. This is somewhat surprising considering genome analyses of these organisms revealed genes encoding for the canonical sulfate reduction pathway, although they cannot perform sulfate reduction (Finster *et al*., 2013; Mardanov *et al*., 2016). Nevertheless, for the disproportionation of thiosulfate and sulfite, biochemical analyses suggested they use pre‐existing enzyme systems typically used for reducing other sulfur compounds, while enzymes involved in elemental sulfur transformations are yet to be revealed (Finster, 2008). Together, these findings highlight the versatility of individual microorganisms to use various sulfur compounds and in differing ways under varied environmental conditions. In this way, the disproportionation of SCI could be an especially useful strategy to switch to when other electron donors and acceptors are limited. Additionally, this must be taken into account when interpreting the presence/expression of functional genes, which are often taken as straightforward indications for, e.g. sulfate reduction, since the individual canonical enzymes in sulfate reduction can be used for various dissimilating metabolisms.

## Organo‐sulfur molecule transformations

Another important, yet largely overlooked component of the marine sediment sulfur cycle involves the utilisation and formation of organo‐sulfur molecules (OSM) (Fig. 1). OSM may include common cellular constituents such as cysteine, methionine, co‐enzymes and co‐factors, which together constitute a relatively small component of organic matter and will not be described further here, while other groups that constitute a greater proportion of organic matter include sulfonates (R‐ ${\text{SO}}_{3}^{–}$) and ‘sulfated’ molecules i.e. sulfate esters/‐O‐sulfonates (R‐O‐ ${\text{SO}}_{3}^{–}$). Together, OSM represent the second most important reduced sulfur pool in marine sediment environments, accounting for up to 35–80% of the reduced sulfur (Bruchert and Pratt, 1996; Passier *et al*., 1999). Sulfonated organics may comprise 20–40% of the organo‐sulfur in marine sediments (Vairavamurthy *et al*., 1994). Although no such studies have been conducted in marine sediments, studies in soils with isotopically labelled sulfur ‘tracers’ show constant fluxes of sulfur in and out of organic molecule pools, and these fluxes are thought to be primarily mediated by microorganisms (Ghani *et al*., 1993aa,b). The cleavage of sulfur moieties from organics by desulfonation may release oxidised sulfur compounds such as sulfite or sulfate, which can be either assimilated, converted and excreted, or utilised as electron acceptors by anaerobic microorganisms. Importantly, desulfonation may enable further catabolic degradation of the organic molecules, thereby allowing them to be used as nutrient and energy sources. This may therefore have important implications as to whether or not such organic matter is mineralised or buried. An immense diversity of sulfated organic molecules are known to exist in nature, including sulfated derivatives of major organic molecule classes such as (poly)saccharides, lipids, aminoglycans, polyaromatics, flavanoids and steroids (Barbeyron *et al*., 2016). A variety of microorganisms are known to desulfonate various organics via different enzymes (Table 1). For instance, members of the marine genus *Rhodopirellula* (phylum *Planctomycetes*) are desulfonating, polysaccharide‐degraders with a remarkable number (up to 110) sulfatase genes in their genomes, which appear to reflect a highly diverse substrate range (Glöckner *et al*., 2003; Wegner *et al*., 2013). Examples of sulfated polysaccharides include animal‐derived chondroitin sulfate, seaweed‐derived carrageenans, and algae‐derived fucoidan. High copy numbers of sulfatases appears to be a trait relatively widespread in the PVC‐superphylum (*Planctomycetes*–*Verrucomicrobia*–*Chlamydia‐Lentisphaerae‐Omnitrophica*) (Thrash *et al*., 2010). Intriguingly, genome comparisons among marine versus freshwater strains of the *Verrucomicrobia* suggest expansions of sulfatase genes in marine strains, likely reflecting the prevalence of these organic molecules in marine environments (Spring *et al*., 2016). Much that is known about desulfonating microorganisms in marine systems comes from aerobes, while the prevalence of these capabilities in anoxic marine sediments is essentially unknown. However, some SRM have the capacity to liberate sulfite from the small molecular weight compound taurine for anaerobic respiration (Lie *et al*., 1999). It is unknown if microorganisms are also able to desulfonate higher molecular weight compounds that may form during diagenetic reactions during sediment burial.

Indications for the ecological significance of OSM in marine environments is reflected in the genomes of microorganisms, whereby genes encoding enzymes for desulfonation of organics to enable further catabolism of the substrates appear to be common in pelagic and benthic marine bacteria (Glöckner *et al*., 2003; Woebken *et al*., 2007; Quaiser *et al*., 2011; Teeling *et al*., 2012). Additionally, our own examinations (unpublished data) of available subsurface *Planctomycetes* and *Bacteroidetes* genomes from tidal flat sediments (Baker *et al*., 2015), also uncovered that these taxa are enriched in sulfatase genes (up to 56 copies in one genome) and may be specialised for the degradation of sulfated organics in anoxic subsurface sediments.

Recent research is beginning to shed light on the importance of the diagenetic sulfurization of organics by reactive sulfides and other SCI produced by sulfur‐cycling microorganisms. Although not directly related to microbial physiology, it may have a large yet overlooked influence on ecology and biogeochemistry. Evidence from recent experiments with humic acid‐like substances at lower pH has shown reactions of sulfide with organic matter may be on par with re‐oxidation with oxygen or iron oxides (Yu *et al.*, 2015). High resolution mass‐spectrometry‐based characterisation of dissolved organic matter in marine sediments has also directly shown a significant proportion of naturally occurring organic matter in sulfidic marine sediments can be sulfurized during early diagenesis, thereby increasing the molecular weight and diversity of the organic matter (Schmidt *et al*., 2017). This therefore means that the activity of SRM has a significant but little acknowledged chemical impact on the molecular structure of organic matter in marine sediments. This may also therefore pertain to the questions of whether or not certain organics are mineralised or buried, since changes in the molecular structure of organic molecules from sulfurization may mean they become unrecognisable to typical enzymes used to degrade the unmodified molecules. Interestingly, it could also be the case that some microorganisms have evolved enzymes to specially attack or dump electrons to such molecular bonds/structures, although these are completely unknown. These little studied aspects underline the highly interlinked nature of the carbon and sulfur cycles, and interesting future lines of research may relate to determining the degradability of such diagenetically‐formed OSM.

## Concluding remarks

Contemporary research has revealed that remarkably diverse microorganisms have evolved to fulfil an array of niches in marine sediments by transforming sulfur compounds among various redox states (Fig. 1). It can therefore be asserted that sulfur is a major evolutionary and ecological driver of microbial life in the seafloor. From the recent deciphering of the molecular underpinnings of key biochemical pathways for fundamentally important sulfur metabolisms such as sulfate reduction, to developing understandings of the various highly interlinked biogeochemical implications associated with the sulfur cycle, we have now acquired a solid grasp about the importance of the sulfur cycle in one of the world's largest biospheres, i.e. the marine sedimentary seabed. While we have come a long way in developing this understanding, it becomes obvious that a great deal of exciting discoveries await to be made in the future that will greatly enhance our understanding of how sulfur sustains and influences microbial life in the marine seabed. We predict several major research avenues for the future are ripe for investigation. Prolific advances in linking molecular biological approaches such as strain‐resolution genome reconstruction from metagenomes, single‐cell genomics, and other molecular ‘omics’ technologies with biogeochemical and physiological processes are an obvious avenue where major progress in understanding the true diversity and revealing the metabolic capabilities of sulfur‐transforming microorganisms can be foreseen. Discovery and description of unknown sulfur‐metabolizing biochemical pathways, e.g. responsible for sulfate reduction in anaerobic methane‐oxidising archaea (Milucka *et al*., 2012), will be an important requirement for improved interpretation of ‘sulfur microbiomics’ data from environmental surveys. Research into the ecological and biogeochemical significance of the diverse, free‐living SOM populations will provide important perspectives on their individual environmental contributions. Additionally, exploration of SCI‐ and organo‐sulfur‐metabolizing microorganisms is needed to fully understand their diversity, ecological niches, and significance in the cycling of sulfur and other elements.
